# Pulmonary Mucormycosis Presenting as Rapidly Progressive Cavitary Pneumonia and Lung Abscess in a Patient With Type 2 Diabetes Mellitus and a Solitary Functioning Kidney: A Report of a Rare Case

**DOI:** 10.7759/cureus.107813

**Published:** 2026-04-27

**Authors:** Rumanas Lafeer, Pakkiyaretnam Mayurathan

**Affiliations:** 1 Internal Medicine, Batticaloa Teaching Hospital, Batticaloa, LKA; 2 University Medical Unit, Batticaloa Teaching Hospital, Batticaloa, LKA

**Keywords:** amphotericin b toxicity, angioinvasive fungal infection, cavitary pneumonia, diabetes mellitus, invasive fungal infection, lung abscess, necrotizing pneumonia, pulmonary mucormycosis, rhizopus species, solitary kidney

## Abstract

Pulmonary mucormycosis is an invasive fungal infection that can present with rapidly progressive pulmonary disease and may mimic bacterial pneumonia or lung abscess. This diagnostic overlap can lead to delayed recognition and treatment.

We report the case of a 33-year-old woman with type 2 diabetes mellitus and a solitary functioning kidney, who presented with fever, productive cough, pleuritic chest pain, and progressive dyspnea. Serial chest radiographs demonstrated rapid progression of lung lesions from right-sided consolidation to cavitary necrosis with air-fluid level formation, consistent with lung abscess. Despite broad-spectrum antibiotic therapy, clinical and inflammatory markers worsened, prompting bronchoscopy evaluation. Histopathology revealed broad aseptate fungal hyphae with right-angle branching. Fungal culture confirmed *Rhizopus* species, compatible with the diagnosis of pulmonary mucormycosis. The patient was treated with intravenous liposomal amphotericin B for six weeks. The therapy was complicated by amphotericin-induced hypokalemia, hypomagnesemia, and transient liver enzyme derangement, necessitating close monitoring given her solitary functioning kidney. She demonstrated gradual clinical and biochemical improvement with appropriate antifungal therapy.

This case highlights the importance of early suspicion of mucormycosis in diabetic patients with non-resolving pneumonia and underscores the challenges of managing antifungal therapy in patients with limited renal reserve.

## Introduction

Mucormycosis is a fulminant, angioinvasive fungal infection caused by molds of the order Mucorales, with *Rhizopus* species being the commonly implicated pathogen in human disease [1]. These organisms are ubiquitous in the environment and are usually acquired via inhalation of airborne spores. Once embedded in the respiratory tract, they demonstrate a predilection for vascular invasion, culminating in thrombosis, tissue infarction, and progressive necrosis [2].

Pulmonary mucormycosis accounts for the second most frequent clinical form after rhino-orbito-cerebral involvement and is associated with high morbidity and mortality. The impact is more pronounced in patients with poorly controlled diabetes mellitus, hematological malignancies, or other evidence of impaired immunity [3]. Diabetes mellitus, particularly when uncontrolled or associated with ketoacidosis, remains one of the strongest risk factors for mucormycosis [4]. In patients with diabetes mellitus, particularly in the setting of poor glycemic control, multiple pathophysiological mechanisms predispose to mucormycosis. Hyperglycemia impairs neutrophil chemotaxis, phagocytosis, and intracellular killing, thereby weakening innate immune defense. In addition, reduced iron sequestration leads to increased free iron availability, promoting fungal growth. Furthermore, hyperglycemia and elevated iron levels upregulate endothelial glucose-regulated protein 78 (GRP78), facilitating fungal adhesion and angioinvasion. These mechanisms collectively enhance vascular invasion, thrombosis, and tissue necrosis [2,4].

Although pulmonary mucormycosis typically presents with atypical symptoms such as fever, cough, hemoptysis, and pleuritic chest pain, radiological findings also mimic bacterial pneumonia, tuberculosis, or malignancy [5]. Consolidation, nodules, cavitary lesions, and the reverse halo sign have been described [5]. In rare instances, progressive parenchymal necrosis can result in cavitation and development of a lung abscess, leading to diagnostic delay if initially managed as a routine bacterial abscess [5].

Infection due to *Rhizopus* species is identified histopathologically by broad, ribbon-like, aseptate or pauciseptate hyphae with right-angle branching and marked angioinvasion [2]. Definitive diagnosis is based on demonstration of characteristic hyphae in tissue and/or fungal culture establishment [6]. Early diagnosis is crucial because mortality rises significantly with delayed antifungal therapy [3]. Current international guidelines recommend prompt initiation of high-dose liposomal amphotericin B as first-line treatment, along with correction of underlying contributing factors and surgical debridement when feasible [6].

We report a rare case of pulmonary mucormycosis caused by *Rhizopus* species presenting as a lung abscess, highlighting diagnostic challenges, radiologic-pathologic correlation, and the importance of early aggressive management in avoiding fatal outcomes.

The objective of this case report is to highlight the diagnostic challenges of pulmonary mucormycosis presenting as a lung abscess in a patient with diabetes mellitus and to emphasize the importance of early invasive diagnostic evaluation. Additionally, this report aims to illustrate the therapeutic challenges associated with the use of amphotericin B in a patient with a solitary functioning kidney, requiring careful monitoring of renal function and electrolyte balance.

## Case presentation

A 33-year-old woman with a four-year history of type 2 diabetes mellitus and bronchial asthma presented with a one-week history of fever, productive cough with rusty sputum, and progressive shortness of breath. She also complained of right-sided pleuritic chest pain. There were no associated autonomic symptoms. She denied orthopnea, paroxysmal nocturnal dyspnea, dysuria, abdominal pain, lower limb swelling, or weight loss.

Her past medical history was significant for a left solitary functioning kidney following right-sided nephrectomy performed at the age of five years for an unknown indication. She had no known drug or food allergies. She was a teetotaler and denied smoking or illicit drug use.

On examination, she was febrile to touch and mildly pale, without icterus. There was no cervical lymphadenopathy, finger clubbing, or peripheral edema. Her respiratory rate was 22 breaths per minute. Oxygen saturation was 94% on room air. The trachea was central. Right-sided chest expansion was reduced, and breath sounds were diminished over the right hemithorax. Coarse crepitations were heard over the right middle and lower lung zones.

Her blood pressure was 130/80 mmHg, and pulse rate was 126 beats per minute, regular with good volume. Cardiovascular examination revealed dual heart sounds in all four areas, without audible murmurs. Abdominal examination revealed a healed right nephrectomy scar; otherwise, the abdomen was soft and non-tender with no organomegaly. Neurological examination was unremarkable.

**Figure 1:**
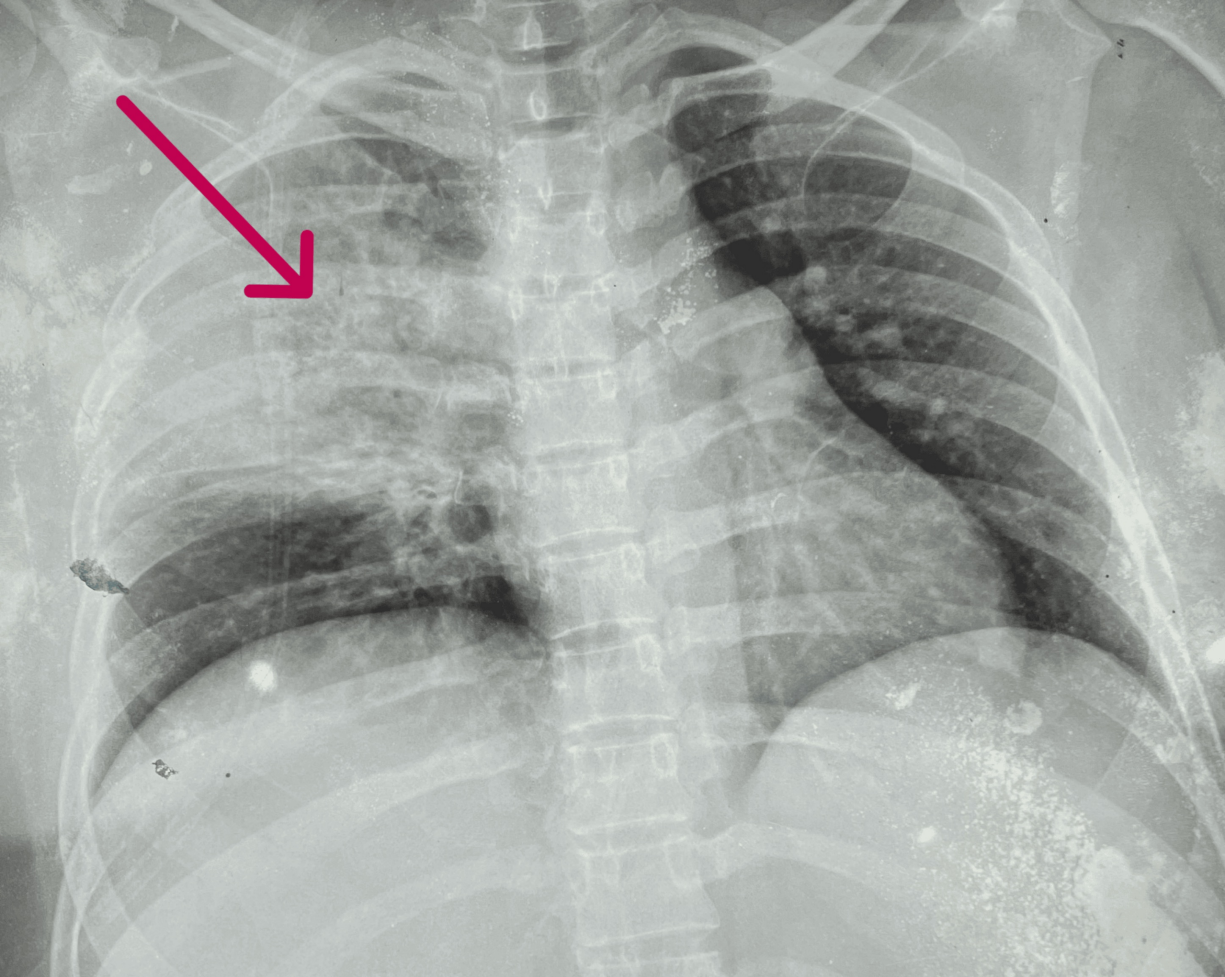
Chest X-ray on admission showing right-sided consolidation (arrow)

Key laboratory investigations during hospitalization are summarized in Table 1. Chest X-ray on admission showed right-sided consolidation (Figure 1).

**Table 1:** Key laboratory investigations during hospitalization WBC: White Blood Cell; CRP: C-reactive Protein; ESR: Erythrocyte Sedimentation Rate; AST: Aspartate Aminotransferase; ALT: Alanine Aminotransferase; Gamma GT: Gamma Glutamyl Transferase; PT: Prothrombin Time; INR: International Normalized Ratio; LDH: Lactate Dehydrogenase

Investigation	Unit	Reference Range	Day 1	Day 3	Day 5	Day 8	Day 12
WBC	×10³/µL	4.0-11.0	11.52	18.99	29.81	24.09	10.9
Neutrophils	×10³/µL	2.0-7.0	8.53	15.51	26.35	26.89	6.88
Hemoglobin	g/dL	12-16	8.1	7.1	6.3	9.6	8.1
Platelets	×10³/µL	150-450	503	480	435	340	321
CRP	mg/L	<5	71	112	200	170	186
ESR	mm/hr	<20	124	-	-	-	-
Creatinine	µmol/L	62-115	143	133	164	125	146
Blood Urea	mmol/L	1.8-6.3	40	5	-	-	-
Sodium	mmol/L	136-145	135	134	140	-	-
Potassium	mmol/L	3.5-5.1	3.8	4.1	2.8	2.6	3.4
Magnesium	mmol/L	0.7-1.0	0.6	0.8	1	-	-
AST	U/L	15-37	108	115	64	-	-
ALT	U/L	12-78	67	64	60	-	-
Alkaline Phosphatase	U/L	46-116	702	430	455	-	-
Gamma GT	U/L	15-85	678	472	472	-	-
Albumin	g/L	34-50	15	15	14	-	-
PT	seconds	11-13.5	16.8	-	-	-	-
INR		0.8-1.2	1.26	-	-	-	-
LDH	U/L	85-227	212	179	235	-	-
Corrected Calcium	mmol/L	2.1-2.5	2.74	-	-	-	-
Urine Protein	g/L	<0.15	3.82	0.31	-	-	-
Protein-Creatinine Ratio	mg/mmol	<15	649	175	-	-	-

**Figure 2:**
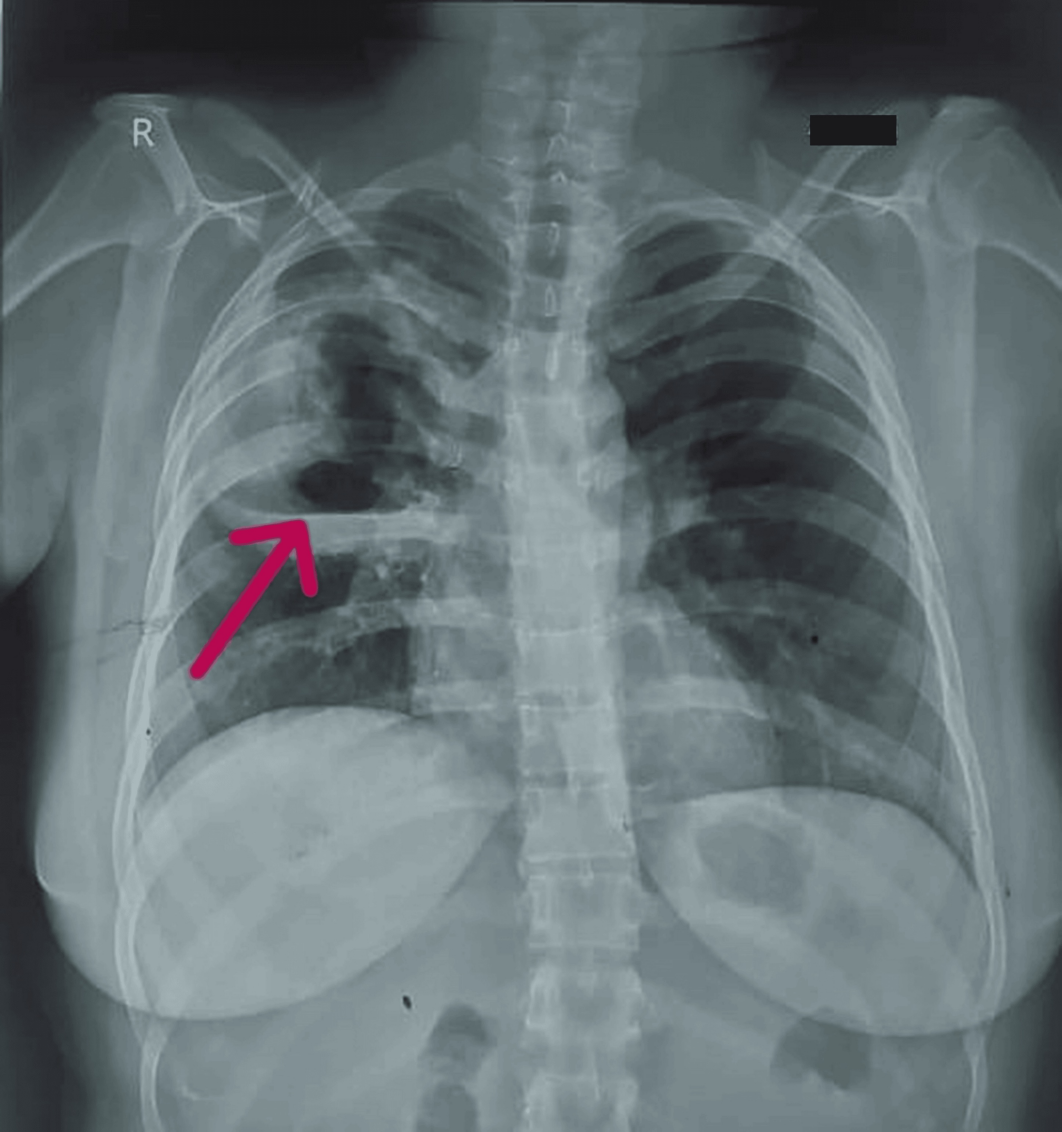
X-ray showing right lung thick-walled cavity lesion (arrow)

Then the following X-ray showed cavity formation (Figure 2).

**Figure 3:**
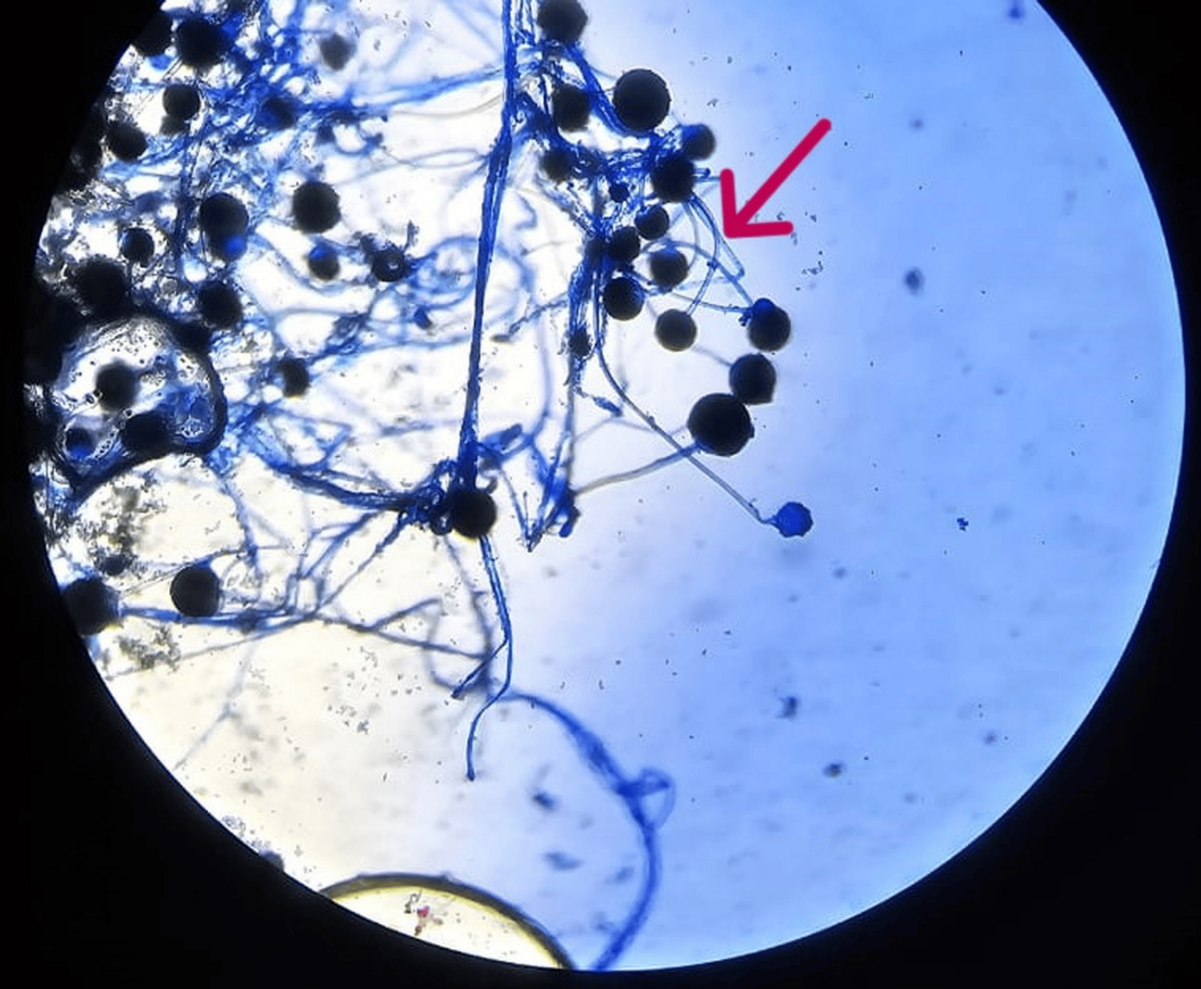
Histopathology image showing aseptate hyphae (arrow)

**Figure 4:**
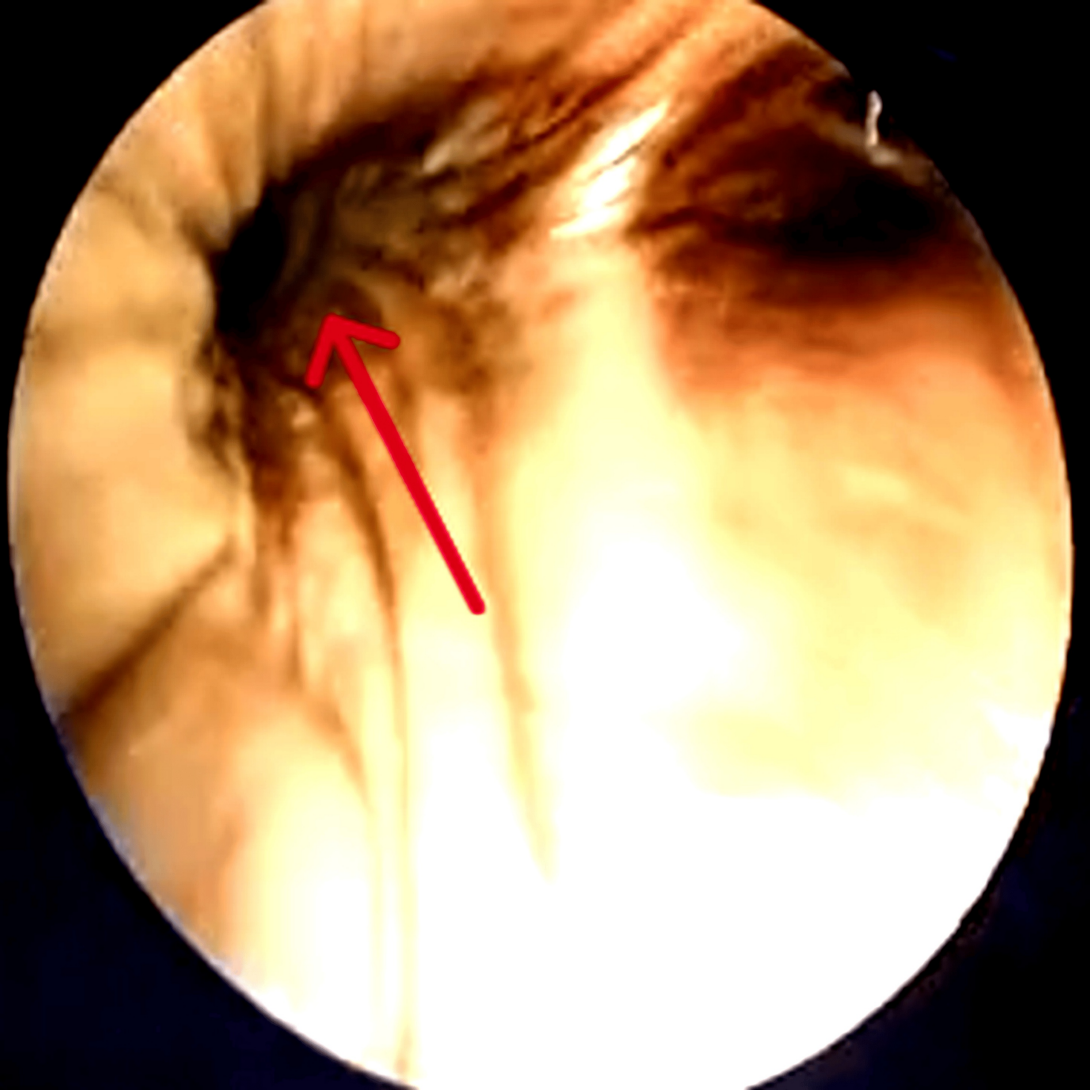
Bronchoscopic image showing suppurative inflammation with associated fungal infection (arrow)

Bronchial wash GeneXpert testing for *Mycobacterium tuberculosis* was negative. Histopathological examination of the bronchial biopsy demonstrated suppurative inflammation with fungal elements (Figure 3). Direct microscopy of the specimen was performed (Figure 4) and demonstrated fungal filaments suggestive of mucormycosis, while fungal culture isolated *Rhizopus* species, confirming the diagnosis of pulmonary mucormycosis. Transthoracic echocardiography demonstrated normal cardiac chamber size and preserved left ventricular systolic function, with no valvular abnormalities or vegetations. Abdominal ultrasound revealed features consistent with acute kidney injury in the solitary functioning kidney. However, the original echocardiography and ultrasound images were not available for inclusion in the manuscript.

The diagnostic process in this patient evolved sequentially. Initially, the clinical presentation and chest radiograph findings were suggestive of bacterial pneumonia, and the patient was initially managed with empirical broad-spectrum intravenous antibiotics, including intravenous ceftriaxone (2 g once daily) and oral clarithromycin (500 mg twice daily), considering a diagnosis of community-acquired pneumonia. However, despite appropriate therapy, the patient demonstrated clinical deterioration with rising inflammatory markers and radiological progression to cavitary necrosis with air-fluid level formation, raising suspicion of a non-bacterial etiology. This prompted further evaluation with bronchoscopy. Histopathological examination revealed broad, aseptate fungal hyphae with right-angle branching, and subsequent fungal culture confirmed *Rhizopus* species, establishing the diagnosis of pulmonary mucormycosis.

Intravenous liposomal amphotericin B was initiated at a dose of 5 mg/kg/day in accordance with international guidelines and continued for six weeks. During therapy, the patient developed significant hypokalemia (nadir 2.6 mmol/L) and hypomagnesemia (0.6 mmol/L), consistent with amphotericin-induced renal tubular toxicity. These electrolyte abnormalities required aggressive replacement and close monitoring. Renal function was carefully observed due to her solitary functioning kidney, and creatinine levels remained stable with supportive management.

Gradual clinical improvement was observed, with defervescence of fever, reduction in inflammatory markers, stabilization of renal parameters, and radiological improvement on follow-up imaging.

## Discussion

Pulmonary mucormycosis is a rapidly progressive angioinvasive fungal infection caused by organisms of the order Mucorales, with *Rhizopus* species accounting for the majority of cases worldwide [1,3]. Diabetes mellitus is considered one of the most important predisposing factors, particularly in patients with uncontrolled diabetes and impaired neutrophil function [4].

The pathogenesis of mucormycosis is characterized by vascular invasion, endothelial damage, and thrombosis, leading to pulmonary infarction and necrosis [2]. This angioinvasive pattern results in the development of cavitary lesions and, in rare circumstances, lung abscess formation, as demonstrated in this case [5]. Lung abscess secondary to mucormycosis is rare and may initially be misattributed to bacterial infection, leading to a delay in appropriate antifungal therapy.

Radiological features are non-specific and include pulmonary consolidation, nodules, cavitation, and the reverse halo sign [5]. However, imaging alone cannot reliably distinguish mucormycosis from other fungal or bacterial causes. That is why tissue diagnosis remains the gold standard [6].

Differentiating pulmonary mucormycosis from bacterial pneumonia is clinically challenging, particularly in the early stages when symptoms and initial radiological findings overlap. In this case, the initial presentation was consistent with bacterial pneumonia; however, the lack of clinical improvement despite appropriate antibiotic therapy, along with rapidly progressive radiological changes from consolidation to cavitary necrosis with air-fluid level formation, raised suspicion of an alternative etiology. These features, together with worsening inflammatory markers, prompted further evaluation with bronchoscopy, leading to a definitive diagnosis. Early recognition of such red flag features is essential to avoid delay in initiating appropriate antifungal therapy.

Histopathological diagnosis of broad, ribbon-like, aseptate hyphae with right-angle branching is characteristic [2]. Isolation of *Rhizopus* species on fungal culture further confirms a definitive diagnosis [6]. Early bronchoscopy played a critical role in establishing the diagnosis, allowing prompt identification after failure of empirical antibacterial therapy.

Mortality rates for pulmonary mucormycosis vary between 40% and 70%, with a higher death rate associated with late diagnosis, disseminated disease, and comorbidities [3]. Prompt initiation of liposomal amphotericin B markedly improves survival outcomes [6].

The therapeutic approach in this case was additionally complicated by the presence of a solitary functioning kidney. Amphotericin B, although potentially lifesaving, is associated with nephrotoxicity and renal tubular electrolyte wasting [7]. The patient developed significant hypokalemia and hypomagnesemia after starting therapy, requiring aggressive correction. Intensive monitoring of renal function and electrolytes was necessary to avoid further renal compromise [7].

This case highlights the importance of maintaining a high index of suspicion for invasive fungal infections in diabetic patients presenting with non-resolving pneumonia, particularly when clinical and radiological deterioration occurs despite appropriate antibacterial therapy. Early bronchoscopy with tissue diagnosis plays a crucial role in establishing the diagnosis and enabling prompt initiation of antifungal treatment, which is essential for improving survival outcomes.

In contrast to many reported cases requiring surgical resection, combined surgical and antifungal management is often recommended in selected cases [8]. However, our patient demonstrated clinical improvement with long-term antifungal therapy alone, highlighting the importance of early detection and multidisciplinary management.

This case highlights the need for a high clinical suspicion of mucormycosis in diabetic patients with poorly resolving pneumonia and reinforces the importance of early invasive diagnostic procedures.

Limitations

This case report has several limitations. As a single-patient report, the findings cannot be generalized to a broader population. Advanced imaging, such as contrast-enhanced CT of the chest, was not available, which may have provided additional radiological characterization of the lesion. Additionally, the absence of echocardiographic and abdominal ultrasound images in the manuscript limits visual correlation with the described findings.

Future research directions

Further studies are required to better characterize the spectrum of pulmonary mucormycosis presenting as cavitary lung disease or lung abscess, particularly in patients with diabetes mellitus. Prospective studies evaluating early diagnostic strategies, including the role of bronchoscopy and advanced imaging, may improve timely diagnosis. Additionally, research focusing on optimizing antifungal therapy in patients with limited renal reserve is warranted to balance efficacy and toxicity.

## Conclusions

Pulmonary mucormycosis is a rapidly progressive and potentially life-threatening angioinvasive fungal infection that may mimic bacterial pneumonia or lung abscess, particularly in patients with uncontrolled diabetes mellitus. Early clinical suspicion and prompt invasive diagnostic evaluation are essential for timely diagnosis. This case illustrates the importance of bronchoscopy with histopathological examination and fungal culture in establishing the diagnosis of pulmonary mucormycosis. Early initiation of liposomal amphotericin B resulted in clinical improvement in our patient despite the presence of a solitary functioning kidney. Careful monitoring of renal function and electrolyte disturbances is crucial during amphotericin therapy.

Early recognition and multidisciplinary management are key to improving outcomes in this severe infection. This case reinforces the need for early consideration of invasive fungal infections in non-resolving pneumonia and highlights the importance of timely intervention to improve clinical outcomes.
